# Unidirectional thermal expansion in edge-sharing BO_4_ tetrahedra contained KZnB_3_O_6_

**DOI:** 10.1038/srep10996

**Published:** 2015-06-05

**Authors:** Yanfang Lou, Dandan Li, Zhilin Li, Shifeng Jin, Xiaolong Chen

**Affiliations:** 1 Research & Development Center for Functional Crystals, Beijing National Laboratory for Condensed Matter Physics, Institute of Physics, Chinese Academy of Sciences, Beijing 100190, China; 2 Collaborative Innovation Center of Quantum Matter, Beijing 100190, China

## Abstract

Borates are among a class of compounds that exhibit rich structural diversity and find wide applications. The formation of edge-sharing (*es*-) BO_4_ tetrahedra is extremely unfavored according to Pauling’s third and fourth rules. However, as the first and the only *es-*borate obtained under ambient pressure, *es*-KZnB_3_O_6_ shows an unexpected high thermal stability up to its melting point. The origin of this extraordinary stability is still unclear. Here, we report a novel property in KZnB_3_O_6_: unidirectional thermal expansion, which plays a role in preserving *es-*BO_4_ from disassociation at elevated temperatures. It is found that this unusual thermal behavior originates from cooperative rotations of rigid groups B_6_O_12_ and Zn_2_O_6_, driven by anharmonic thermal vibrations of K atoms. Furthermore, a detailed calculation of phonon dispersion in association with this unidirectional expansion predicts the melting initiates with the breakage of the link between BO_3_ and *es-*BO_4_. These findings will broaden our knowledge of the relationship between structure and property and may find applications in future.

It is well established that anion polyhedra of small and high-valence cations are connected to each other via corner sharing in crystalline matters as postulated in Pauling’s third and fourth rules so as to minimize the Coulomb repulsion among cations. The validity of these rules is confirmed by a vast number of borates, silicates and phosphates as well. Exceptions are only met in borates synthesized under high pressures (HP) and high temperatures by Huppertz and coworkers recently. Typical compounds are Dy_4_B_6_O_15_^1^, α-RE_2_B_4_O_9_ (RE = Eu, Gd, Tb, Dy)^2^, RE_4_B_6_O_15_ (RE = Dy, Ho)^3^ and HP-NiB_2_O_4_^4^. A common feature in these borates is that they all contain BO_4_ tetrahedra connected to each other through edges instead of corners as in more than 1000 borate structures determined over the past decades. High pressure is essential in forming edge-sharing (*es-*) BO_4_ tetrehedra and even in inducing a conversion from BO_3_ planar triangle to BO_4_ tetrahedra^5^. But it is generally accepted that the pressure-driven formation of *es-*BO_4_ compounds is not thermodynamically favored; they are metastable over their ambient counterparts.

More recently, Chen’s group^6^ and Wu’s group^7^ independently reported the synthesis of a new borate KZnB_3_O_6_ under ambient pressure. This is the first borate with *es-*BO_4_ obtained without the aid of high pressures. Examination of its crystal structure reveals that BO_4_ tetrahedra in KZnB_3_O_6_ are nearly identical in B-O bond lengths and O-B-O angles to the previously reported HP borates^1^,^2^,^3^,^4^. First principles calculations^6^ show that *es-*KZnB_3_O_6_ is indeed more energy-favored than the hypothetical corner-sharing (*cs-*) one, which was constructed based on isostructural KCdB_3_O_6_^8^. Yang and coworkers^9^ made a comparative study of *es-* and *cs-* KZnB_3_O_6_ by lattice dynamics and electronic-structure calculations and showed a soft-mode exists in the *cs-*isomorph, probably due to an overlong Zn-O bond in ZnO_5_ polyhedra, while there is none in the *es-*one. A study by nuclear magnetic resonance excludes the B-B bond^10^ in BO_4_. The origin, however, responsible for the formation of *es-*BO_4_ in *es-*KZnB_3_O_6_ in an energy-favorable way still remains unclear.

An interesting property in this newly-discovered KZnB_3_O_6_ is that its structure can be well preserved from room temperature up close to its melting point 1073 K, without any detectable phase transition occurring. This suggests that the structure or at least the fundamental building block (FBB) is very rigid to thermal attack. As is well-known, the way by which crystalline materials respond to changes in temperature offers a direct and straightforward measure of the nature of chemical bonds and structural peculiarities. In this work, we present the results of a study of the thermal expansions of KZnB_3_O_6_ from room temperature to 1013 K. It is found that unidirectional thermal expansion along the approximate [-3 0 2] direction exists over the entire measured temperature range from 298 K to 1013 K, which can fully accounts for the total volume thermal expansion; meanwhile, the expansions along other directions on the plane perpendicular to [-3 0 2] are negligibly small, i.e. the area shows zero expansion. By analyzing the variations in bond lengths, we show that the *es-*BO_4_ is virtually immune to changes in temperature thanks to the hinge rotations of B_6_O_12_ and Zn_2_O_6_ rigid groups, which only leads to a quasi-unidirectional expansion upon heating. The phonon dispersions and partial density of states (PDOS) calculations reveal a soft mode at 1013 K, due to transverse vibrations of the bridge oxygen whereby BO_3_ and B_2_O_6_ are connected, develops to cause the structure to dismantle at around its melting point. These results shed light on why KZnB_3_O_6_ can be stabilized over such a wide temperature range.

## Results

**Figure 1:**
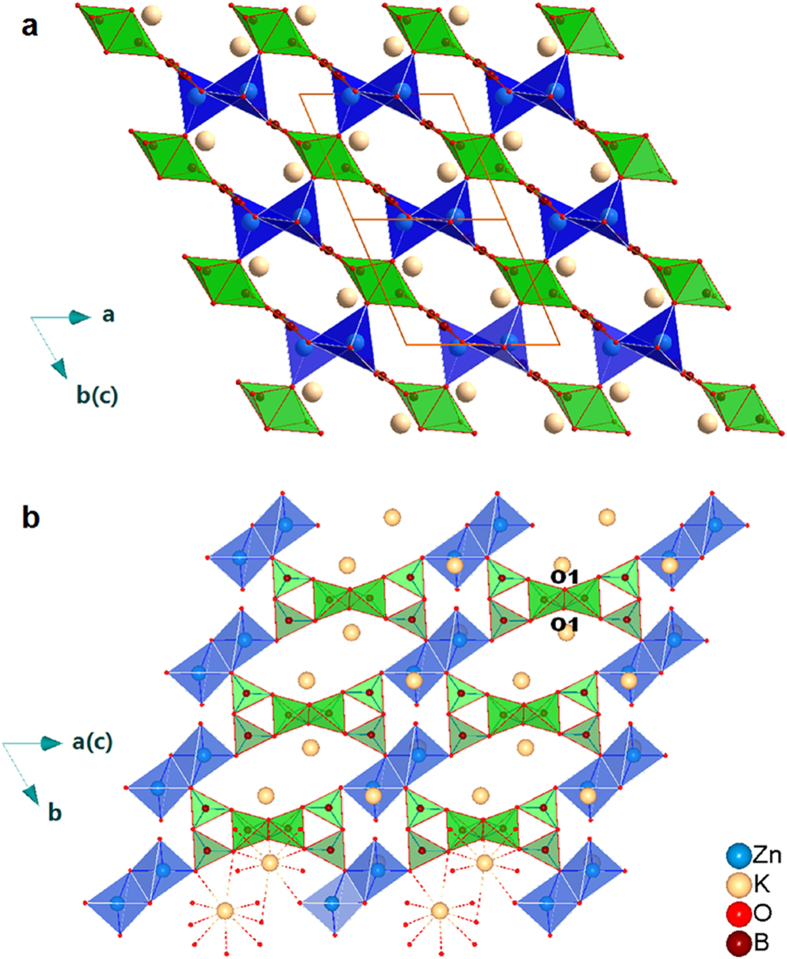
Structure of KZnB_3_O_6_. (**a**) Polyhedral view of the KZnB_3_O_6_ crystal structure projected along the [01–1] direction, with tetrahedra ZnO_4_ in blue, triangular BO_3_ and tetrahedra BO_4_ units in green, adapted from Fig. 1a) in Ref.6. (**b**) Connection details of B_6_O_12_ and Zn_2_O_6_ blocks in the (-111) plane. The metal-borate framework is built up from BO_3_ triangles, BO_4_ tetrahedra, ZnO_4_ tetrahedra. The FBB consists of two *es-*BO_4_ tetrahedral and four vertexes-shared BO_3_ triangles.

KZnB_3_O_6_ powder samples were synthesized through solid state reactions from A.R. K_2_CO_3_, H_3_BO_3_ and ZnO. The preparing details can be consulted elsewhere^6^ or in Methods Section. This compound crystallizes in a triclinic unit cell with lattice constants a = 6.753 Å, b = 6.911 Å, c = 7.045 Å, α = 63.39 ^o^, β = 72.584 ^o^, γ = 69.13 ^o^ and space group P-1. Figure 1a shows the polyhedral view of its crystal structure, in which the metal-borate framework is built up from BO_3_ triangles, BO_4_ tetrahedra, ZnO_4_ tetrahedra. The FBB, B_6_O_12_ block, consists of two BO_4_ tetrahedra and four BO_3_ triangles. The BO_4_ tetrahedra are O1-O1 edge shared to each other and further corner-shared by BO_3_ trangles in their outer vertex O, see Fig. 1b. Two ZnO_4_ tetrahedra form a Zn_2_O_6_ polyhedron also through edge-sharing. Each FBB is bonded by six Zn_2_O_6_. K fills the voids left by these polyhedra.

**Figure 2:**
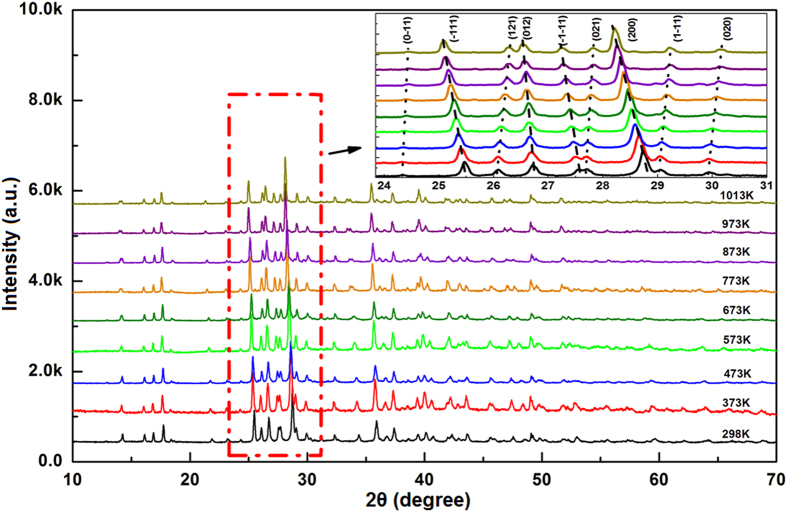
The powder X-ray diffraction patterns collected at 298 K, 373 K, 473 K, 573 K, 673 K, 773 K, 873 K, 973 K and 1013 K for KZnB_3_O_6_, respectively. The inset is a closer view of the enlargement of 2θ = 24 ^o^ ~ 31 ^o^ portion indicates that some peaks shift obviously towards low-angles, others nearly unshift, still others slightly towards high angles.

**Figure 3:**
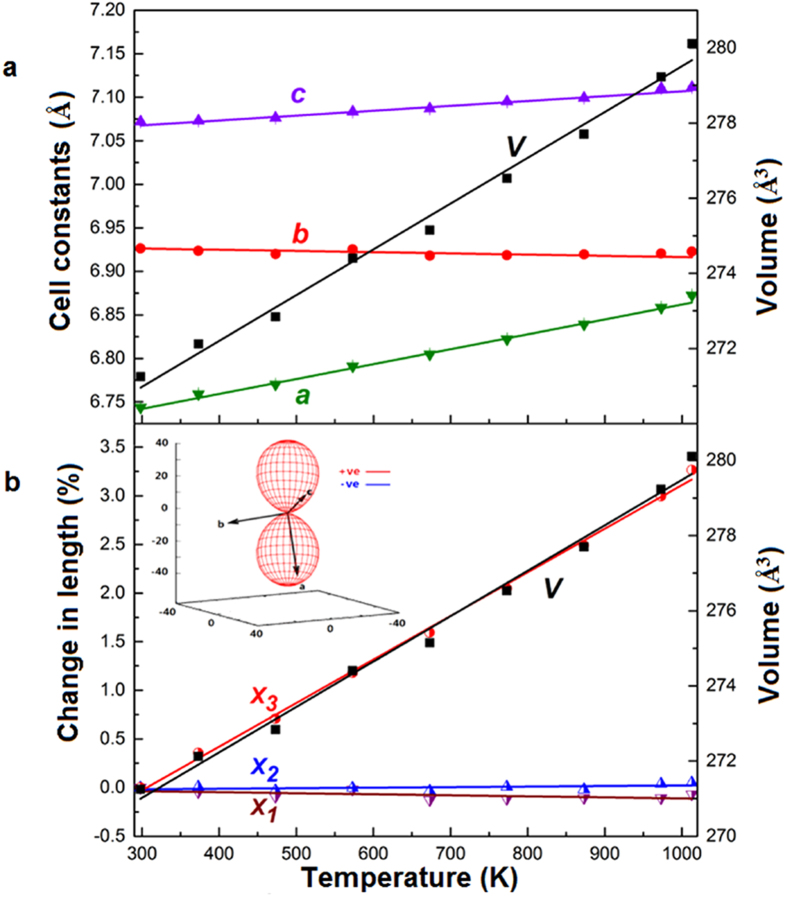
Thermal expansion behavior of the KZnB_3_O_6_. (**a**) The temperature dependence of lattice constants a, b, c and cell volume. (**b**) Normalized components of the principal axes versus temperature, where X_1_, X_2_ and X_3_ are (−1.06 ± 0.42) MK^−1^, (0.56 ± 0.31) MK^−1^ and (44.81 ± 0.79) MK^−1^, determined by the PASCal^14^ program. Inset: The thermal expansivity indicatrix, which describes the expansion rate in all directions in the set of orthogonal axes. It clearly shows the area of zero thermal expansion and a very large expansion along the X_3_ axis.

Figure 2 shows the powder X-ray diffraction patterns collected at 298 K, 373 K, 473 K, 573 K, 673 K, 773 K, 873 K, 973 K and 1013 K for KZnB_3_O_6_, respectively. A closer view of the enlargement of 2θ = 24 ^o ^~ 31 ^o^ portion indicates that some peaks shift obviously towards low-angles, others nearly unshift, still others slightly towards high-angles, reflecting the changes in the unit cell are very anisotropic with increasing temperatures. All peaks can be indexed based on triclinic cells with high figure of merits by using Dicvol06^11^. As an example, the lattice constants for the 298 K pattern are a = 6.744 Å, b = 6.926 Å, c = 7.072 Å, α = 63.13 ^o^, β = 72.40 ^o^, γ = 69.07 ^o^, in good consistency with ones obtained from the single crystal data. No phase transition occurs as no additional peaks emerge or disappear in the entire temperature range measured. The temperature dependent lattice constants a, b, c and cell volume are plotted in Fig. 3a. All the data along with the crystallographic angels are deposited in Supplementary Information, see Supplementary Table S1. We can clearly see lattice constants a and c linearly expand with the increasing temperatures, while b shrinks, resulting in volume expansion at a rate of (44.92 ± 1.5) × 10^−6^ K^−1^. This is an average thermal expansion rate for borates^12^,^13^.

Since the crystallographic axes of the present triclinic unit cell are chosen by convention, the variations in the lattice constants will not always reflect the fundamental thermal response of KZnB_3_O_6_. This is, in particular, true for the low symmetry compounds. It is established that a unique set of orthogonal axes, or the principal axes exist, along which the expansion or contraction describes the fundamental thermal response, including some nonzero shear components, or rather, the variation in the crystallographic angles of the unit cell for a given compound. To this end, we applied web-based software PASCal^14^ to find the set of orthogonal axes by inputting the unit cell data at all measured temperatures. Figure 3b gives the changes in length along the principal axes with the increasing temperatures. The principal coefficients of thermal expansions, their estimated standard deviations and the components of the principal axis relative to the crystallographic axes are summarized in Table 1. In comparison with the expansion data for the crystallographic axes, the variations of the principal axes with increasing temperatures are more anisotropic, the expansion rates for X_1_ and X_2_ being (−1.06 ± 0.42) × 10^−6^ K^−1^ and (0.56 ± 0.31) × 10^−6^ K^−1^, respectively, much smaller than one for the X_3_, (44.81 ± 0.79) × 10^−6^ K^−1^. Two facts are evident. First, the area expansion rate for the plane determined by X_1_ and X_2_ is virtually zero as α_area_ = α_x1 _+ α_x2_ = (−0.5 ± 0.42) × 10^−6^ K^−1^. Second, the expansion rate for X_3_ is nearly equal to the volume expansion rate, α_x3 _≈ α_v _≈ 45 × 10^−6^ K^−1^. These facts suggest the volume expansion can be ascribed to the expansion occurring in the X_3_ axis, which is along approximate [-3 0 2] in the crystallographic coordinates. The inset of Fig. 3b is the thermal expansivity indicatrix which describes the expansion rates in all directions in Cartesian coordinate. It clearly shows an area zero thermal expansion and a very large expansion along the X_3_ axis. Although it is a common phenomenon that borates have large anisotropic thermal expansion^12^,^13^, such as recently-reported LiBeBO_3_ which shows an area negative expansion^15^ from 193 K to 273 K, the quasi-unidirectional thermal expansion for KZnB_3_O_6_ in such a wide temperature range is very rare and peculiar^13^,^16^,^17^.

## Discussion

**Figure 4:**
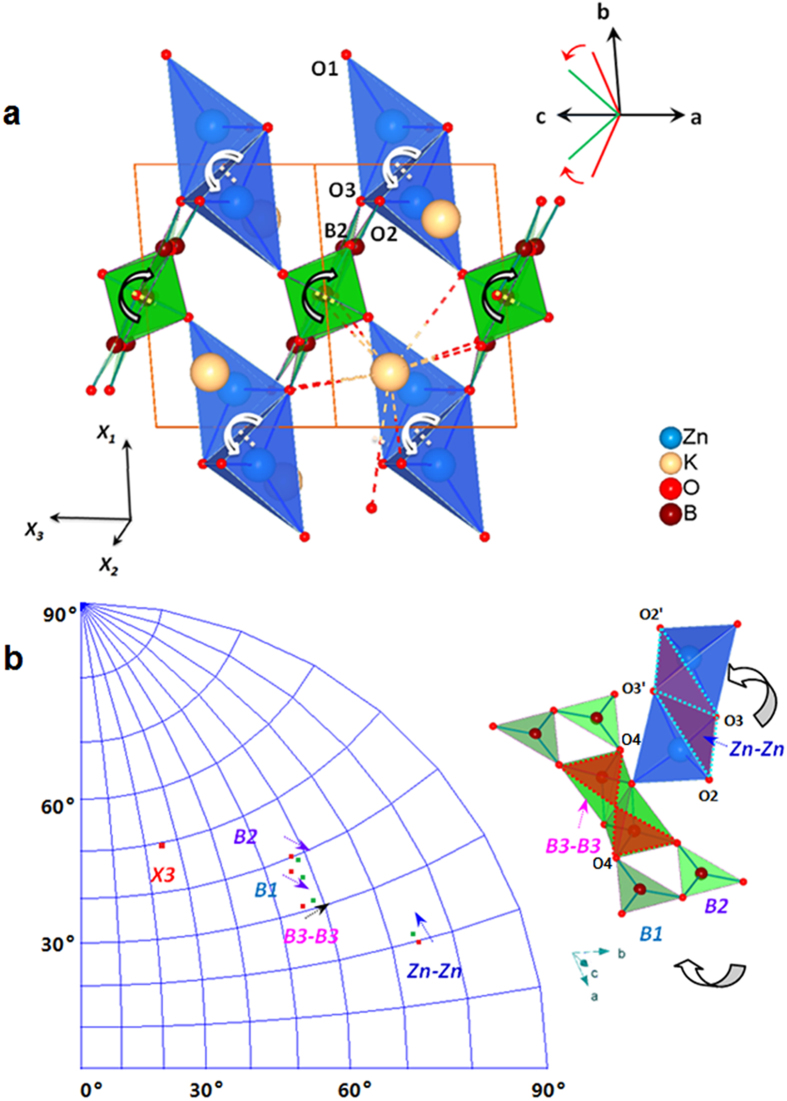
Hinge rotation of the KZnB_3_O_6_. (**a**) The rigid groups’ rotations associate with the area zero thermal expansion. Top right: schematic of ‘hinge folding’ that simultaneously expands in a,c and contracts in b. (**b**) Left: The stereographic projection of B3-B3, B1, B2 and Zn-Zn planes at 298 K and 1073 K, rotations of those planes with increasing temperatures are indicated by arrows. Right: ‘hinge’ rotation of the contacted FBB and Zn_2_O_6_ units with temperature, where B3-B3 plane is defined by four O atoms bridging the BO_4_ and BO_3_ atoms, B1 and B2 planes are defined by two BO_3_ triangles, Zn-Zn plane is the brown plane defined by O2, O3, O2’ and O3’.

To understand the peculiar thermal response and gain some insights on the thermal stability for KZnB_3_O_6_, we performed the geometry optimization and lattice dynamics study by density functional theory (DFT) calculations. We mimic the high temperatures by using the experimental lattice constants at high temperatures as constraints while allowing the geometry optimization of the atomic positions in the unit cell and subsequent lattice dynamics calculations. The calculating details and the results are archived in Supplementary Information. Careful examination of the bond lengths at 298 K and 1013 K reveals that the expansions in B-O and Zn-O bonds are generally below 0.5%, while up to 3.4% in K-O bonds (see Supplementary Table S2). Moreover, the Hirshfeld’s “rigid body” test^18^,^19^,^20^,^21^,^22^,^23^ indicated that the maximum differences in MSDA’s (mean square displacements of atoms) have magnitudes ∆ = 15 Å^2^ in group B_6_O_12_, ∆ = 9 Å^2^ in group Zn_2_O_6_. Meanwhile the maximum value is as large as ∆ = 108 Å^2^ for KO_9_ group (Table S3). It is then reasonable to take Zn_2_O_6_ and B_6_O_12_ as rigid groups and KO_9_ as an easily-deformable polyhedron in the temperature range from 298 K to 1013 K. We first consider the rigid rotations of bodies since they play a major role in negative- or zero-thermal expansions for compounds that contain rigid tetrahedra and octahedra^24^,^25^,^26^,^27^, etc., including B-O polyhedra^12^,^13^,^28^. For KZnB_3_O_6_, the rotations of rigid groups are shown in Fig. 4a. Reverse rotations are identified for B_6_O_12_ and Zn_2_O_6_ along the [1 0 1] direction, see Fig. 4a. Taking the angle between the normals of plane B3-B3 and Zn-Zn (see Figure caption for the definition of these planes) as an indicator for the relative rotation, it decreases by about 2.71 ^o^ from 298 K to 1013 K. These rotations can be better viewed in Fig. 4b, where the planes B2, B1 and B3-B3 generally rotate in an opposite way as the Zn-Zn plane does, for the angles between normals of the corresponding plane become smaller. For K-O bonds, we note that their elongations vary a lot, from 0.19% to 3.39%. The concerted effect of reverse rotations of rigid groups and very asymmetrical elongations in K-O bonds lead to expanding along roughly [–3 0 2], i.e., the X_3_ axis direction, leaving the other two directions, or rather, the plane perpendicular to [–3 0 2] almost unchanged upon heating (Supplementary Fig. S1). In addition, the FBB is found to become more flat with increasing temperatures. It can be seen that the angles between the B1, B2 and the B3-B3 planes decrease simultaneously, suggesting a reduced geometry distortion within the FBB upon heating. We think that these unusual thermal responses help secure the *es-*BO_4_ from disassociation.

Furthermore, the inversion symmetry of KZnB_3_O_6_ puts some limits on the deformation of B_2_O_6_. Since two B3 and two O1 are connected each other by –1, respectively, their only possible change in positions are either closer or away along their co-axial directions. Lengthening O1-O1 is unlikely because this kind of expansion will be inhibited by increasing B3-B3 repulsion as they become closer. Instead, the shortening O1-O1 is energy favored. Similar situation is for Zn_2_O_6_ polyhedra. This may explain why the *es-*BO_4_ polyhedra are stable at high temperatures.

The calculated phonon dispersions and PDOS at 298 K and 1013 K are shown in Supplementary Fig. S2. Most of vibrational branches shift towards low frequency when temperature increases from 298 K to 1013 K. Correspondingly, the PDOS at the low frequency band are enhanced. This can be better viewed from Supplementary Fig. S3, which shows the PDOS contributed by O4 for example. A few branches, however, shift a little or even towards high frequencies as see the PDOS contributed from O2. These results are consistent with our observations that KZnB_3_O_6_ expands in a very anisotropic way, large expansitivity along X_3_, zero along X_1_ and X_2_. Moreover, we find that an optical mode becomes negative at the Γ point in the Brillouin zone at 1013 K, implying a structural instability at this temperature. Meanwhile, the calculated vibration amplitudes associated with the negative optical mode reveal that the largest one resides on O4, which connects the BO_3_ triangles and the BO_4_ tetrahedra, see Supplementary Fig. S4. Accordingly, we speculate that the melting begins with the breakage of the link between BO_3_ and BO_4_ when temperature is close to its melting point.

Thermal responses of materials have been a hot research topic over the last decades. Many a compound was found to exhibit negative thermal expansions, others area negative expansions. The unidirectional thermal expansion reported here for KZnB_3_O_6_ is rarely known^13^,^29^. This unusual thermal response is related to the peculiar structure. This property may find future applications in its single crystal form in fields that require a very small change in diameters while expanding in the third dimension over a wide temperature range, such as a high precision optical lens.

In summary, the first borate with edge-sharing BO_4_ synthesized under ambient pressure, KZnB_3_O_6_, exhibits an unusual unidirectional thermal expansion: 45 × 10^−6^ K^−1^ with zero thermal expansion in the perpendicular direction over a temperature range from room temperature to 1013 K. The volume expansion can be regarded as coming from an expansion in one direction only. We determine that this unusual thermal behavior comes from rotations of rigid groups of B_6_O_12_ and Zn_2_O_6_, probably driven by very asymmetrical elongations of K-O bonds. In this way, the *es-*BO_4_ is secured from disassociation upon heating. Based on the present study, KZnB_3_O_6_ can be described as a 1-dimensional compound in the sense of thermal expansion. Other related properties of this compound merit further investigation.

## Methods

### Samples

The compound KZnB_3_O_6_ was prepared by grinding a mixture of K_2_CO_3_ (A.R.), ZnO (A.R.), and H_3_BO_3_ (99.99%) to a fine powder, and then heating at 500 °C for 12 h to decompose the salt. In order to compensate for volatilization of alkali metals, 15% excess of K_2_CO_3_ (A.R.) is required. The sample was reground and annealed at 750 °C for 24 h, and a single-phase white powder was readily obtained. The sample purity was verified by X-ray powder diffraction.

### High temperature XRD measurements

Temperature-dependent *in situ* X-ray diffractometry was performed on an XPERT-PRO powder diffractometer system (CuKα1; 1.54056 Å) equipped with an Anton Paar HTK-1200N Oven Sample stage. The room-temperature diffraction pattern in the angular range from 10 ^o^ to 80 ^o^ with a scanning step width of 0.017 ^o^ was firstly obtained as a standard, and then the sample was heated from 373 K to 1013 K at intervals of 100 K. Each diffraction pattern was obtained 30 min after the required temperature was reached. Unit cell parameters were then calculated by using the pattern indexing software Dicvol06^11^.

## Additional Information

**How to cite this article**: Lou, Y. *et al.* Unidirectional thermal expansion in edge-sharing BO_4_ tetrahedra contained KZnB_3_O_6_. *Sci. Rep.* **5**, 10996; doi: 10.1038/srep10996 (2015).

## Supplementary Material

Supplementary Information

## Figures and Tables

**Table 1:** The expansivity, estimated standard deviation from room temperature to 1013 K for KZnB_3_O_6_ and the components of principal axes relative to the crystallgraphic axes.

**Axes**	**α(MK**^**−1**^)	**δα(MK**^**−1**^)	**a**	**b**	**c**
X1	−1.056	0.415	0.112	−0.167	0.980
X2	0.561	0.313	0.453	−0.331	0.828
X3	44.809	0.789	−0.835	0.023	0.550
V	44.920	1.528			
