# Supplementary material for: Unidirectional thermal expansion in edge-sharing BO4 tetrahedra contained KZnB3O6
Source: Sci Rep. 2015 Jun 5;5:10996. doi: 10.1038/srep10996 (PMC4457155; doi:10.1038/srep10996)
Supplement: Supplementary Information [file srep10996-s1.pdf]

Supplementary Information

Unidirectional thermal expansion in edge-sharing BO_4 tetrahedra contained $KZnB_3O_6$

Yanfang Lou[1], Dandan Li[1], Zhilin Li[1], Shifeng Jin[1, *], Xiaolong Chen [1,2, *]

[1]Research & Development Center for Functional Crystals, Beijing National Laboratory for Condensed Matter Physics, Institute of Physics, Chinese Academy of Sciences, Beijing 100190, China.

[2]Collaborative Innovation Center of Quantum Matter, Beijing 100190, China.

Correspondence and requests for materials should be addressed to S.F.J (email: shifengjin@iphy.ac.cn) or to X.L.C (email: chenx29@iphy.ac.cn).

Computational methods

The first-principles calculations presented in our work were performed with the CASTEP program code with the plane-wave pseudopotential method[1]. We adopted the generalized gradient approximation (GGA) in the form of the Perdew-Burke-Ernzerhof for the exchange-correlation potentials[2]. The ultrasoft pseudopotential with a plane-wave energy cutoff of 380 eV and a 4×4×4 Monkhorst Pack k-point mesh in the reciprocal space were used for all the calculations[3]. The self-consistent field was set as 5×10^{-7} eV/atom. Based on the experimental lattice parameters, all independent internal atomic coordinates were optimized with the convergence standard given as follows: energy change less than 5×10^{-6} eV/atom, residual force less than 0.01eV/Å, stress less than 0.02GPa, and displacement of atom less than 5×10^{-4} Å. The phonon frequencies and phonon density of states (PHDOS) were obtained with the finite displacement method based on the optimized structures.

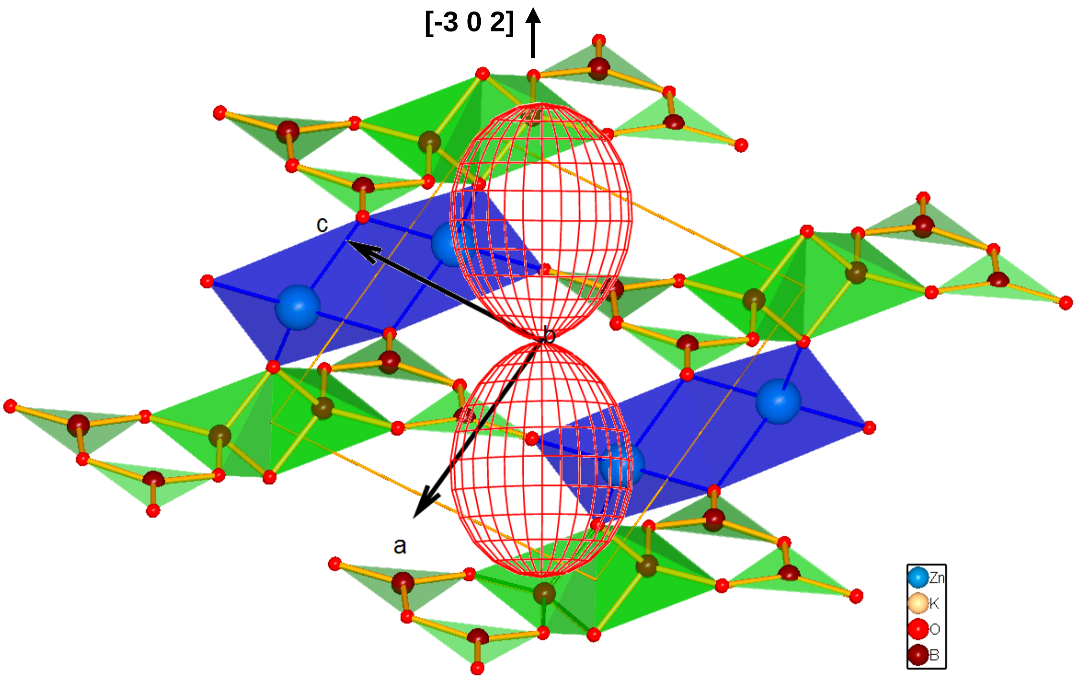

Supplementary Figure S1 | The thermal expansion pole figure comparing with crystal structure.

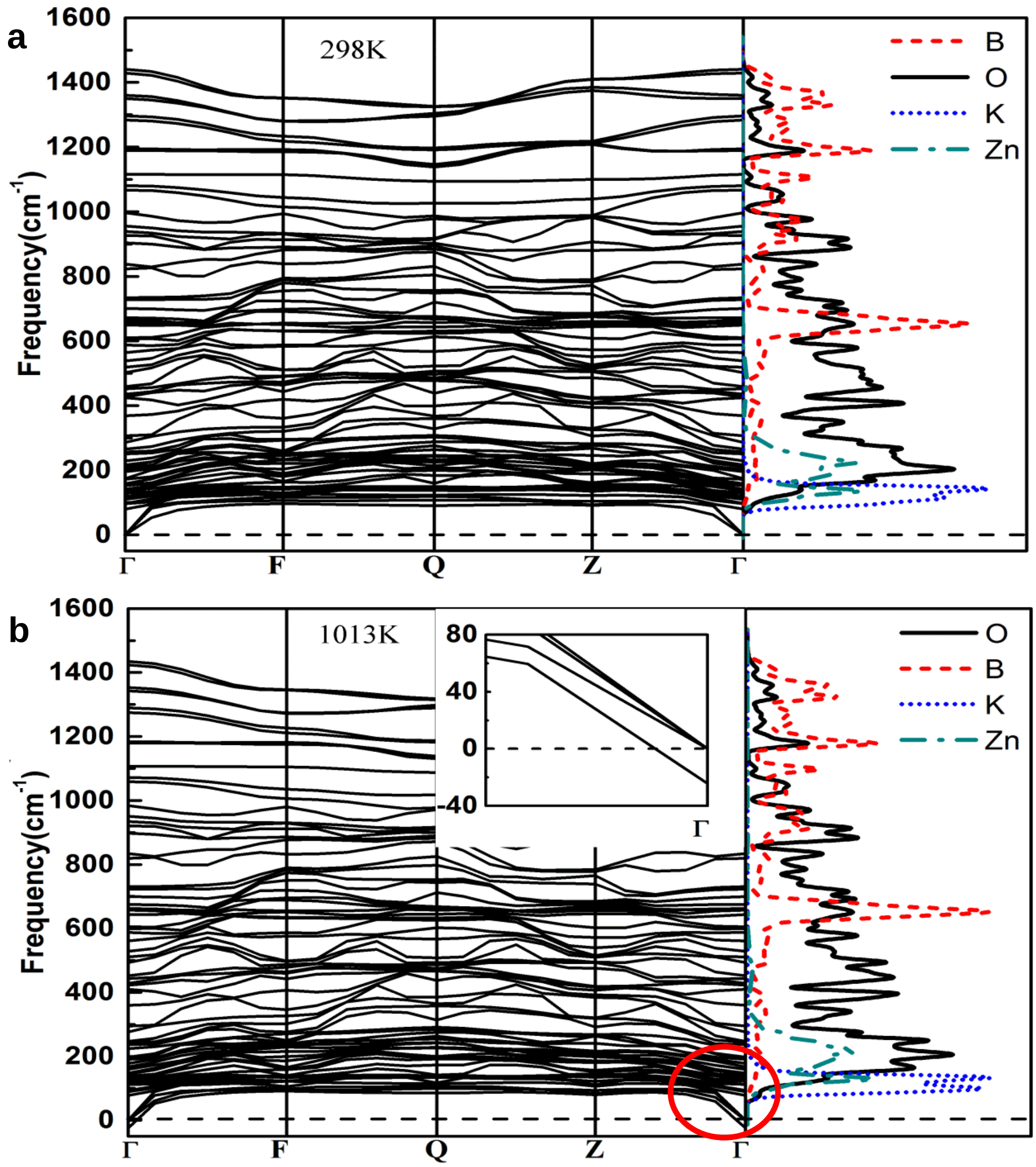

Supplementary Figure S2│ Phonon dispersion and partial phonon DOS for $KZnB_3O_6$ at (a) 298K and (b) 1013K. Inset of Fig. (**b**) shows dispersion curves in the red ellipse, where an optical mode becomes negative at the Γpoint in the Brillouin zone.

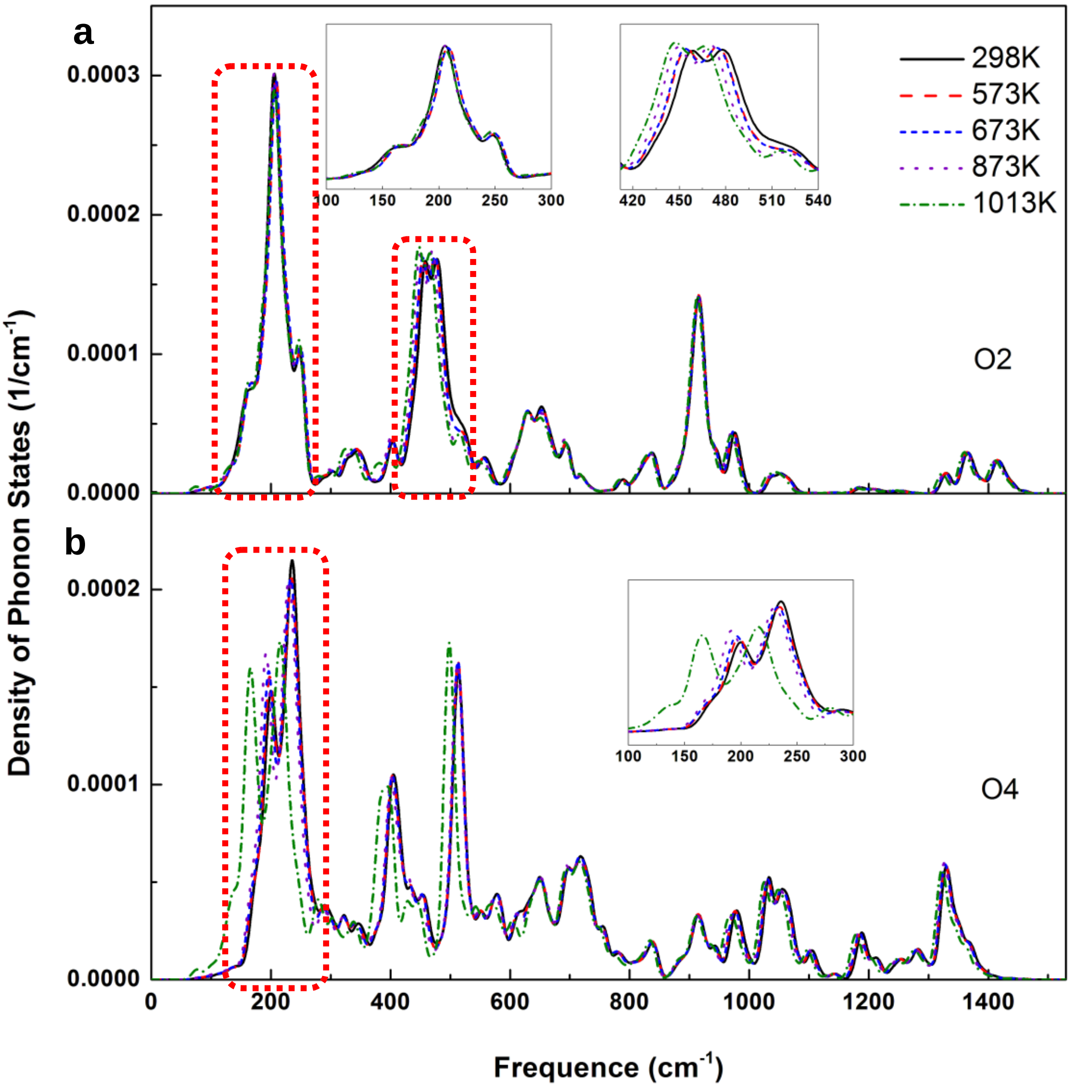

Supplementary Figure S3│ The PDOS for (a) O2 (b) O4 at 298K, 573K, 673K, 873K and 1013K. The insets show enlarged PDOS in the red rectangles.

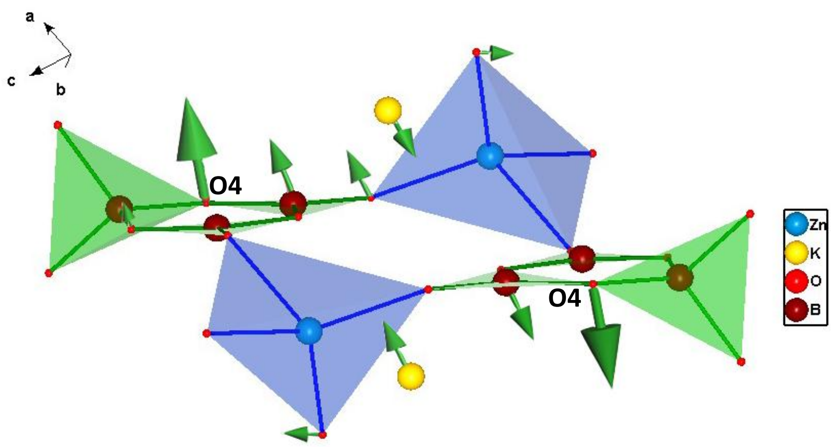

Supplementary Figure S4│ The calculated atomic vibrational mode corresponding to the imaginary optical mode at 1013K. The vibration amplitudes (length of arrows) reveal that the largest one resides on O4, which connects the BO_3 triangles and the BO_4 tetrahedra.

Supplementary Table S1│ The Variation of lattice constants versus temperatures for $KZnB_3O_6$

Temperature(K)	a (Å)	b (Å)	c (Å)	α (°)	β (°)	γ (°)	V (Å^3)
298	6.744(1)	6.926(1)	7.072(1)	63.13(1)	72.40(1)	69.07(1)	271.25
373	6.759(3)	6.924(1)	7.073(1)	63.17(2)	72.56(3)	69.13(2)	272.12
473	6.770(1)	6.920(1)	7.076(1)	63.184(3)	72.764(4)	69.167(4)	272.84
573	6.791(1)	6.925(1)	7.084(1)	63.19(1)	72.96(1)	69.19(1)	274.4
673	6.805(1)	6.918(1)	7.087(1)	63.223(4)	73.20(1)	69.25(1)	275.15
773	6.822(1)	6.919(1)	7.095(1)	63.26(1)	73.41(1)	69.30(1)	276.53
873	6.839(1)	6.921(1)	7.099(1)	63.26(1)	73.63(1)	69.36(1)	277.70
973	6.858(2)	6.921(2)	7.110(1)	63.26(1)	73.86(2)	69.41(2)	279.23
1013	6.873(2)	6.923(2)	7.112(1)	63.29(1)	73.96(2)	69.42(2)	280.11

Supplementary Table S2│ The variation of bond lengths at 298K and 1013K

Bonds	Bonds length at 298K(Å)	Bonds length at 1013K(Å)	The proportion of varitation
K-O1	2.777	2.831	1.95%
K-O2	2.615	2.646	1.16%
K-O2	3.130	3.209	2.52%
K-O3	2.919	3.018	3.39%
K-O4	2.866	2.878	0.45%
K-O5	2.796	2.845	1.76%
K-O5	3.145	3.184	1.24%
K-O6	2.840	2.867	0.97%
K-O6	2.839	2.844	0.19%
Zn-O1	1.949	1.956	0.35%
Zn-O2	1.901	1.905	0.21%
Zn-O3	1.974	1.984	0.53%
Zn-O3	2.062	2.060	-0.07%
B1-O2	1.339	1.340	0.07%
B1-O5	1.383	1.384	0.07%
B1-O6	1.415	1.418	0.20%
B2-O3	1.362	1.363	0.10%
B2-O4	1.368	1.369	0.07%
B2-O6	1.404	1.407	0.16%
B3-O1	1.499	1.503	0.28%
B3-O1	1.516	1.516	0.03%
B3-O4	1.455	1.457	0.12%
B3-O5	1.452	1.454	0.12%

Supplementary Table S3│ The differences in MSDA's (mean square displacements of atoms) from Zn_2O_6, B_6O_{12} and KO_9

[Symmetry codes: (i) -x, -y,-z]

Hirshfeld test	
Bond	$\Delta Uij \times 10^4$ (Å^2)
Zn- O1^i	6
Zn- O2	3
Zn- O3	9
Zn- O3^i	8
B3- O1^i	5
B3- O5	15
B3- O1	3
B3- O4	6
B1- O2	13
B1- O5	9
B1- O6	10
B2- O3	2
B2- O6	14
B2- O4	9
K- O2	108
K- O4^i	54
K- O5	22
K- O6	19
K- O1	8
K- O2^i	27
K- O3^i	15
K- O5^i	81
K- O6^i	3

1. Clark, S. J. *et al*. First principles methods using CASTEP. *Z. Kristallogr.* **220,** 567-570 (2005).

2. Perdew, J. P., Burke, K., Ernzerhof, M. Generalized Gradient Approximation Made Simple. *Phys. Rev. Lett.* **77,** 3865-3868 (1996).

3. Monkhorst, H. J., Pack, J. D. Special points for Brillouin-zone integrations. *Phys. Rev. B* **13,** 5188-5192 (1976).
